# A conversational agent system for dietary supplements use

**DOI:** 10.1186/s12911-022-01888-5

**Published:** 2022-07-07

**Authors:** Esha Singh, Anu Bompelli, Ruyuan Wan, Jiang Bian, Serguei Pakhomov, Rui Zhang

**Affiliations:** 1 CSE Department, College of Science and Engineering, University of Minnesota, Minneapolis, MN USA; 2 College of Pharmacy, University of Minnesota, Minneapolis, MN USA; 3 Institute for Health Informatics and College of Pharmacy, University of Minnesota, Minneapolis, MN USA; 4 Health Outcomes and Biomedical Informatics, College of Medicine, University of Florida, Gainesville, FL USA

**Keywords:** Dietary supplements, Question answering, Conversational agent, Natural language processing, Deep learning, Named entity recognition

## Abstract

**Background:**

Dietary supplements (DS) have been widely used by consumers, but the information around the efficacy and safety of DS is disparate or incomplete, thus creating barriers for consumers to find information effectively. Conversational agent (CA) systems have been applied to healthcare domain, but there is no such system to answer consumers regarding DS use, although widespread use of DS. In this study, we develop the first CA system for DS use.

**Methods:**

Our CA system for DS use developed on the MindMeld framework, consists of three components: question understanding, DS knowledge base, and answer generation. We collected and annotated 1509 questions to develop a natural language understanding module (e.g., question type classifier, named entity recognizer) which was then integrated into MindMeld framework. CA then queries the DS knowledge base (i.e., iDISK) and generates answers using rule-based slot filling techniques. We evaluated the algorithms of each component and the CA system as a whole.

**Results:**

CNN is the best question classifier with an F1 score of 0.81, and CRF is the best named entity recognizer with an F1 score of 0.87. The system achieves an overall accuracy of 81% and an average score of 1.82 with succ@3 + score of 76.2% and succ@2 + of 66% approximately.

**Conclusion:**

This study develops the first CA system for DS use using the MindMeld framework and iDISK domain knowledge base.

**Supplementary Information:**

The online version contains supplementary material available at 10.1186/s12911-022-01888-5.

## Introduction

The utilization of DS (e.g., vitamins, minerals, botanical extracts, and protein powders) in the United States (US) has dramatically increased in recent years. The 2019 Council for Responsible Nutrition (CRN) survey shows that 77% of US adults take DS and 87% (5% increase in 2 years) express overall confidence in the safety, quality, and effectiveness of DS [1]. The utilization of DS does not require a prescription, and consumers usually find health information about DS use by searching the internet themselves. Many sites contain basic facts about DS, their therapeutic use, safety warnings, effectiveness, and information on DS-related research studies. However, a lot of this online DS information is of low quality; and sources of this information are also heterogeneous in nature, ranging from health organizations, government agencies, universities, interest groups, and lay consumers. The distribution of DS information across various DS resources (e.g., Natural Medicines [2], Memorial Sloan Kettering Cancer Center [3]) was found fragmented and inconsistent. Further, the knowledge representations used in these DS resources vary from unstructured free text to more structured searchable data. While these databases or resources provide basic DS knowledge, they either contain incomplete information or lack a standardized knowledge representation (e.g., not using standardized terms for adverse events) that allows these resources to be integrated into a more comprehensive DS KB. Recently, we have developed an integrated and standard DS knowledge base (i.e., iDISK [4]), which can facilitate efficient and meaningful dissemination of DS knowledge. In the study, our system is developed with the assistance of iDISK as the DS knowledge base in the backend.

There is extensive prior work on natural language understanding and answering consumer questions regarding various health-related issues [5–7] and a number of automated online and offline biomedical conversational systems exist [8–13]. Recent development and advances in voice recognition, natural language processing (NLP), and artificial intelligence (AI) have led to the increasing availability and use of conversational agents (CA)-any dialogue system that not only conducts NLP but also responds automatically using human language [14]. Thus, CA's have increasingly become an integral part of our day-to-day lives. CA systems could be classified into 4 types: (a) interaction mode, (b) chatbot application, (c) rule-based AI, (d) domain specific or open domain [15]. Based on their goals, CAs can be categorized into two main types (a) task-oriented and (b) non-task-oriented [16–18]. Recent work on CAs has focused on personalization of CA [19] as well as CA applications in specific domains [20]. There has also been some work on CAs in the biomedical domain [21–23] with one of the most recent publications by Dina et al. [24].

To the best of our knowledge, there is no prior study on the development of a CA system for DS consumers. Thus, the objective of this study is to develop a DS-focused CA system to answer questions related to DS use. We also evaluated the feasibility of using the domain knowledge base (i.e., iDISK [4]) and the MindMeld framework for developing the CA system. Thus, our paper describes a task-oriented and domain-specific CA system.

## Methodology

**Fig. 1:**
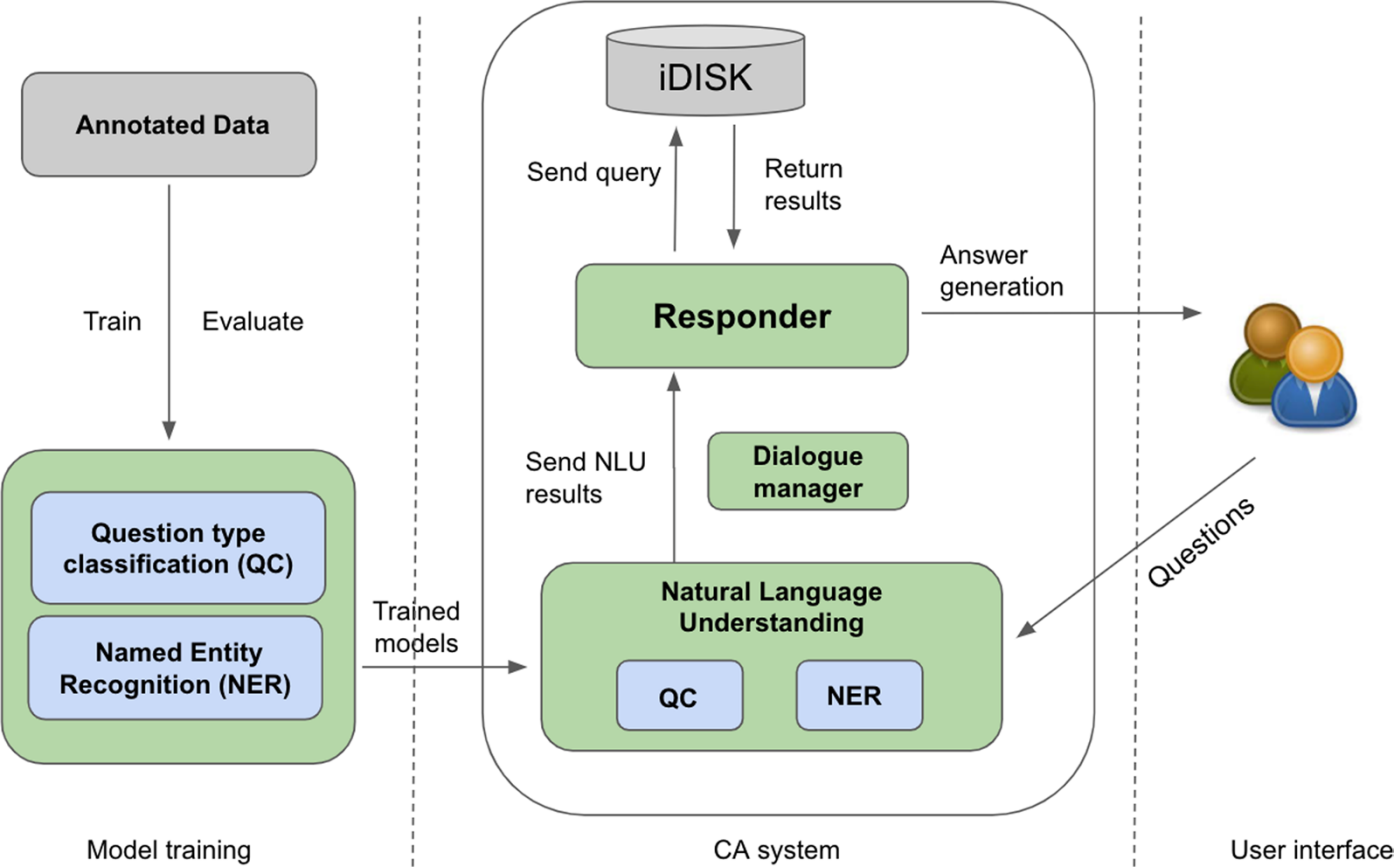
CA system architecture. NLU, natural language understanding

The system architecture and the details of specific processes in each step of its development are shown in Fig. 1. We also discuss the evaluation process for our CA system. In the following sections, we described (1) the CA system architecture (2) dataset and annotation, (3) knowledge base, (4) methods for understanding users’ questions, (5) answer generation, and (6) evaluation of the CA system.

### CA system architecture

We chose the MindMeld as our CA system platform. MindMeld is very well designed for task-driven CA applications and has a set of robust utilities for deploying CA applications as a web service. Compared to other CA systems (e.g., Dialogflow requires sending user data to a third-party server), MindMeld provides robust and fully open-source toolkits that can be deployed as a standalone service. Although MindMeld provides various functionality for conversational flow, this study utilizes only its dialogue manager functionality. The remaining components like natural language processing and question-answering capabilities are replaced with custom-built modules (that is the Natural Language Understanding [NLU] component of our CA system). This is done because MindMeld provides limited options with respect to NLU sub-modules for downstream tasks. For example, MindMeld does not provide CNN or BERT-based model options. The following sections will describe details of the dataset and method development.

The CA system architecture consists of 3 following components:

- The first component as shown in Fig. 1 below consists of a question understanding module which has 2 sub-modules—question type classifier and named entity recognizer (NER).
- The second component is the DS knowledge base-iDISK. We utilize this to provide fast reliable answers to user queries.
- The third component is answer generation. We use MindMeld [25] as our base CA framework and within it, we have one sub-module for answer retrieval, that queries the “Knowledge Base” (KB)—iDISK. This KB is part of our CA framework and contains data extracted from iDISK obtained after per- forming various transformation and pre-processing steps. Thus, it is analogous to a secondary database or cached data and helps for faster information retrieval.

### Dataset and annotation

**Fig. 2:**
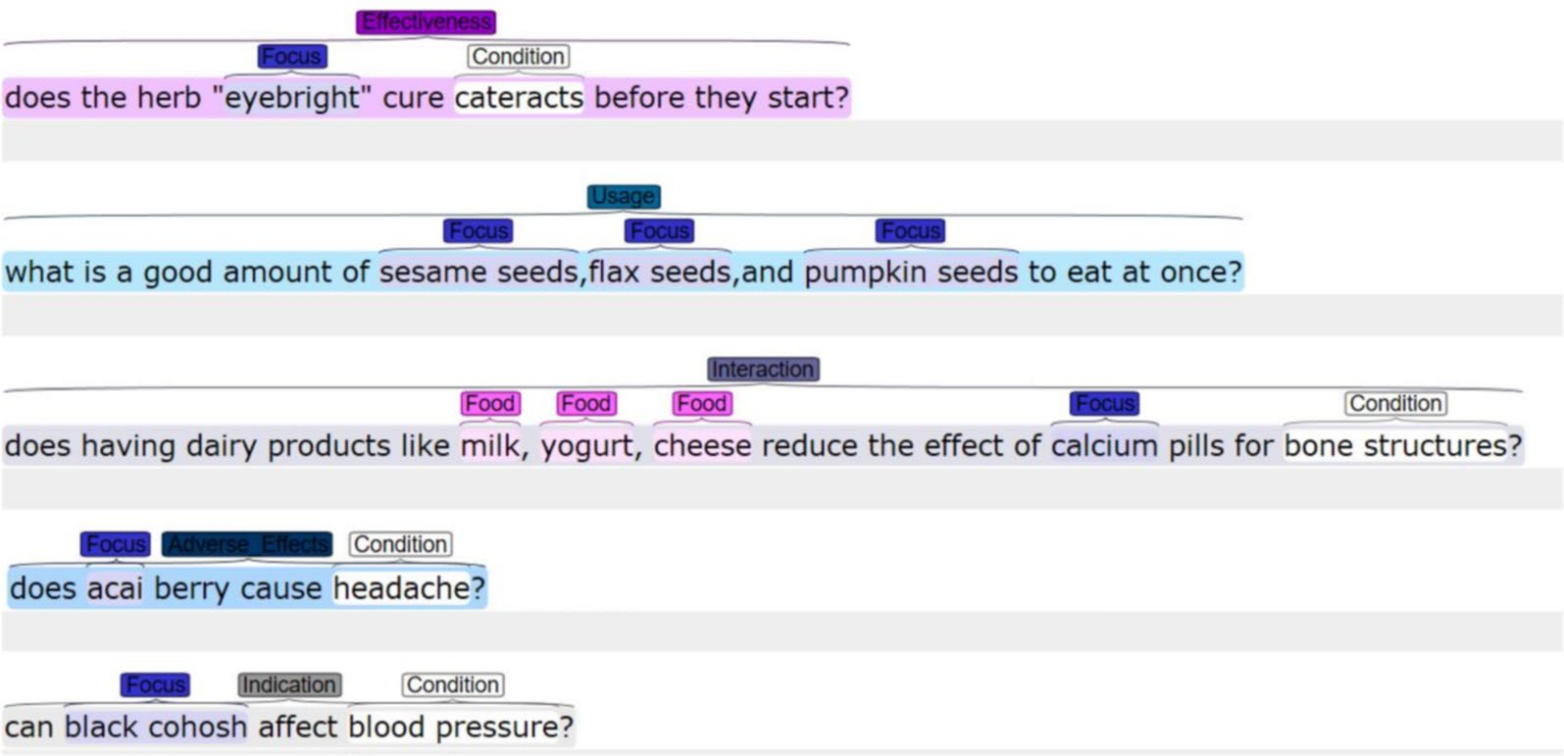
Few examples to demonstrate our manual question annotation

Dataset was accrued by extracting questions (including their titles and contents) from one of the sub-categories “Alternative Medicine” in Yahoo! Answers [26]. We randomly extracted 2000 questions from Yahoo! Answers corpora and manually annotated these samples. Initially, three annotators (led by a pharmacy graduate) manually annotated a random set of 100 questions to determine the annotation guideline to determine the question focus and question type information. They all annotated another set of 100 questions and the inter-annotator agreement was as- sessed using Fleiss’s kappa [27] resulting in a score of 0.86. The annotators discussed the discrepancies until all disagreements were solved. Further, they independently annotated the rest questions of the corpus. However while annotating, questions that were irrelevant and not complete were excluded. A final corpus consists of 1509 questions with annotated question focus, question type, and named entities. Figure 2 depicts a few examples with our manual annotation structure.

### Question understanding

Question understanding is the most essential component for developing our system. Towards question understanding with respect to the domain of DS, there are 2 main tasks-understanding what type of questions are being asked and recognizing all relevant named entities that the user asks about. The first task can be formulated as question type classification whereas the second task can be viewed as a NER problem.

### Question classification

For this classification task, classes are the 8 question types. In this study, we bench-marked 3 machine learning models and 4 deep learning models for question classification using our annotated data. The machine learning models include Random Forest as a baseline, support vector machine (SVM), and logistic regression. Sparse vector representations for every word are obtained using GloVe embeddings [28] (840B tokens, 2.2M vocab, cased, 300 dimensions). A comprehensive evaluation of the explored methods is described in the“Answer Generation" below.

Among deep learning methods, we experimented with Long-Short Term Memory (LSTM), Bi-LSTM, Bi-GRU with attention, and Convolutional Neural Network (CNN) models [29]. The CNN model consists of 4 layers-an embedding layer, a convolutional layer, pooling layers, and a full-connected layer with softmax. Vector representations for every word in the sentence are obtained using Glove embeddings. Varied filter sizes of (1,2,3,5) are applied to the convolutional layer followed by the max-pooling layer which is further used to extract the most important or relevant features generated from the convolution layer. The max-pooling scores from each filter were concatenated to form a single vector, which goes through a dropout and is fed into a fully connected layer. LSTM, Bi-LSTM, and Bi-GRU are all shallow networks with only 4 layers. The last layer for all the 3 methods is a fully-connected layer with softmax activation. More experimental details are available in Additional file: 1.

### Named entity recognition

After identifying the question type for a user query, it is essential to recognise what relevant named entities are present in the query. These named entities along with question type guide the answer generation process. The annotated dataset includes 4 types of entities-Dietary Supplement (DS), Disease (DIS), Medication (MED), and Miscellaneous (MISC). These samples were parsed using Spacy dependency parsing [30] and following BIO [31] tags schema.

For the NER task, we experimented with BERT [32] and BiLSTM-CRF [33] including a linear statistical model-Hidden Markov Model (HMM) [34]. BiLSTM-CRF based methods are standard for NER task. This network can efficiently use past and future input features via a Bi-LSTM layer and sentence level tag information via a CRF layer. A CRF layer is represented by lines that connect consecutive output layers. But recently BERT was among state-of-art methods for the NER task. But for this study, considering the limited dataset size, a simpler model would intuitively be a better choice. This reasoning was also aided by two other factors-faster inference time and class imbalance. Thus, the CRF model outperforms other methods in both these aspects. More experimental details are available in Additional file: 1.

### iDISK knowledge base

User questions around DS are answered by retrieving information from the iDISK [4], which integrates and standardizes DS-related information from 4 existing resources including the NM, the “About Herbs” page on the MSKCC website, the DSLD, and the LNHPD. It consists of 7 concept types (i.e., SDSI: Semantic Dietary Supplement Ingredient, DSP: Dietary Supplement Product, DIS: Disease or Syndrome, SPD: Pharmaceutical Drug, SOC: System Organ Class, SS: Sign or Symptom, TC: Therapeutic Class) and 6 relations between these concept types (i.e., ‘SDSI has adverse effect SOC’, ‘SDSI has adverse reaction SS’, ‘DSP has ingredient SDSI’, ‘SDSI has therapeutic class TC’, ‘SDSI interacts with SPD’, ‘SDSI is effective for DIS’), where the relations entails different relationships between these concept types. iDISK is a graphical database and the current version of iDISK contains 4,208 DS concepts, which are linked via 6 relationship types to 495 drugs, 776 diseases, 985 symptoms, 605 therapeutic classes, 17 system organ classes, and 137,568 DS products. As it is stored in the structured triple (subject-relation-object), so it is easy to retrieve the answer by inputting the subject/object (entities in the questions) and relation (inferred by a question type). It is publicly available through this link.

**Fig. 3:**
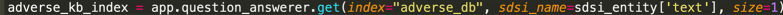
Example search API query structure [36]

To answer DS-related questions, we exploit iDISK knowledge base. This ensures a faster and more accurate domain-oriented query resolution as compared to open-domain answering using the web or search engines. There are various transformations that are performed on our database iDISK and these transformations are stored as JSON objects to create MindMeld databases. Search API queries are built to search the knowledge base using Elasticsearch [35]. The search API queries structure is defined by MindMeld [36] as a part of the QuestionAnswerer Module which by default uses Elasticsearch backend. An example query structure is provided in Fig. 3. Every domain and subsequent intent can use a separate knowledge base. To query a knowledge base, we have to create a corresponding index that represents that database which in-turn can be called by a particular function. Our framework exploits this functionality as we have multiple knowledge base files (JSON) each containing information for every relationship (e.g., has ingredient) or domain that is answerable by iDISK. To understand the transformations it would be beneficial to superficially describe the types of questions that can be answered by our CA. Questions pertaining to (a) any sort of relations, (b) background or generic information, and (c) source information of any DS can be answered. As the questions are diverse within the domain of DS, to answer questions that involve any sort of relationships between two named entities, we perform joins and restrict the number of entries. The join is usually between MRREL.RRF [4] which stores the relation (eg, “interacts with”) and their source information, and MRCONSO.RRF which stores various DS names. Finally, all the data is transformed into JSON format which is fed into our CA framework. As our system also supports one-to-many mapped questions, each entry in the knowledge base can have multiple occurrences in different JSON dumps. Once the appropriate information is retrieved, we route these to appropriate response/answer handlers. These handlers help generate full semantic responses which are in some form of pre-defined templates.

### Answer generation

Results of both question type classification and NER enable us to generate appropriate queries to the knowledge base. The type of question being asked is used to know which relation types to look for and named entities extracted from user queries are used to structure appropriate knowledge-base searches. To generate natural language responses to user requests, once we get the relevant results from the knowledge base, slot-filling style templates are used which are rendered by a “Responder” object [25]. Slot-filling style templates are pre-defined structured sentence templates with multiple blank slots that can be filled with any extracted information on-the-fly. For each of the 8 question-types, we have a pre-defined pseudo-template for example: for Safety question-type, template is: “Here is what I found about {DS}”:

“{DS safety information from iDISK}”. Similarly, for adverse effects question type template used is: The {DS} has Adverse Effects like {adverse effects of DS}. The consecutive answers for these 2 question-types are discussed in the section of “evaluation of responses for user questions” and more examples of such answer generation templates are shown in Table 1.

**Table 1:** Answer generation templates for different question types. The information extracted from our database is substituted in place of curly bracket placeholders

Question type	Answer generation template
Availability	Maybe you can find the information here: {DS^a^ in- formation link 1} or {DS information link 2}
Adverse effects	The {DS} has Adverse Effects like {adverse effects of DS}
Background	{relevant DS background information}
Effectiveness	{DS} is effective for {disease/Symptom}
Indication	{DS} is effective for {disease/Symptom}
Interaction	{DS} interacts with {medication/drug}
Safety	Here is what I found about {DS}: {DS safety information from iDISK}
Usage	This might help: {DS/symptom/medication information link 1} or {DS/symptom/medication information link 2}

^a^Denotes ”Dietary Supplements”

### Evaluation of the CA system

This section details the investigative steps performed for each of the CA system components and their corresponding results. We randomly collected another set of 64 examples in addition to the 1509 samples and were designated to be used as a hold-out test set for end-user evaluation (This set is not a part of the validation samples in previous section). For evaluating our CA’s responses to a user question. Following the LiveQA Track [37]’s (also followed by Dina et. al [24], which simplified the range to a more comprehensive scale) judgment scores on a scale of 1 to 4: 1-incorrect, 2-incorrect but related, 3-correct but incomplete, 4 correct and complete answer. The final results were then transferred to 0–3 range where 0 for poor or unreadable, 1 for fair, 2 for good and 3 for excellent. With this scale we computed two metrics:

- *Average score*: This evaluates the first retrieved answer for every test question (transfers 1–4 level grades to 0–3 scores) [24, 37].
- *succ@i*+ : number of questions with score i or above (where i ranges from 2 to 4) divided by the total number of questions. For example, succ@2 + measures the percent of questions with at least a fair grade answered by the CA [37].

While average score and mean reciprocal rank [38] measure how reliable or fair the answers are (evaluated by humans), we also compute Response Error Rate (RER) [39] as a measure of how coherent and accurate our CA is.

## Results

### Question type annotation dataset

The 8 question types in the annotated corpus include interaction, usage, effective-ness, adverse effects, indication, background, safety, and availability. Table 2 displays the distribution of 8 question types in our annotated dataset. Question type “Effec- tiveness” has the highest number of samples whereas “Safety” has the least. Table 3 lists sample questions with their annotated question type and named entities.

**Table 2:** Distribution of samples for 8 classes in our annotated dataset

Question type	Number of samples
Availability	147
Adverse Effects	133
Background	125
Effectiveness	318
Indication	188
Interaction	237
Safety	108
Usage	253

**Table 3:** Examples of questions in the dataset

Question	Type	Named entity
Does anyone know if you can take ephedrine while taking levothyroxine?	Interaction	Ephedrine, levothyroxine^b^
L-glutamine whats an appropriate dosage for ibs?	Usage	L-glutamine, IBS^a^
Does Niacin really work?	Effectiveness	Niacin
Does acai berry cause headache?	Adverse effects	Acai berry, headache^a^
Does anyone know the health benefits to barley grass?	Indication	Barley grass
What is Milk Thistle?	Background	Milk thistle
Is brewer’s yeast safe?	Safety	Brewer’s yeast
Where can I buy Selenium pills?	Availabilty	Selenium

Each question is annotated for it’s question type and named entity within the question

The column “Named Entity” contains one or two entries. The first entry are entities of type”DS” except the rows marked with^a^ and^b^. The rows with^a^ have a second entity entry of type”DIS” and rows with ^b^have second entry as “MED”

### Evaluation of question understanding module

This section details the results of evaluation on the 2 sub-modules of question understanding-question type classification and NER. Table 4 displays evaluation results of different question classifiers in our question understanding module. The results are reported for 4 metrics - Precision, recall, F1 (all of which are weighted) and accuracy. Our deep learning methods consistently outperform machine learning methods including our baseline model. CNN has the best performance on question classification with a weighted F1 score of 81%. Hence, overall F1 weighted for question classification which is also our standard accuracy is 81%. Table 5 includes results for NER submodule. Vanilla CRF model outperforms the other 3 methods and has F1 weighted of 85%.

**Table 4:** Performance of various question type classifiers

Model	Precision	Recall	F1	Accuracy (%)
Random Forest	0.603	0.556	0.540	55.60
SVM (with RBF Kernel)	0.629	0.629	0.620	62.90
SVM (with Linear Kernel)	0.647	0.649	0.640	64.90
Logistic Regression	0.656	0.649	0.64	64.90
BiGRU + Attention	0.737	0.728	0.729	72.85
LSTM	0.800	0.790	0.790	78.67
Bi-LSTM	0.800	0.790	0.790	79.41
CNN	0.810	0.800	0.810	80.79

**Table 5:** Performance of various entity recognizers

Model	Precision	Recall	F1	Accuracy (%)
BERT	0.629	0.629	0.620	62.90
BiLSTM + CRF	0.723	0.678	0.698	73.20
HMM	0.719	0.820	0.740	82.00
CRF	0.872	0.860	0.850	87.10

### Evaluation of responses for user questions

Table 6 displays a sample of responses from our CA. On our evaluation set of 64 question-answer pairs, we observed an average score of 1.82 which indicates that responses are fair and mostly good. For the succ@i metric, we compute succ@2+ and succ@3+ and observe scores of 0.762 and 0.656 respectively. These scores are consistent with the intuition that as we increment the score range (grade scale 1–4) probability of correct answers decreases. Thus, the system is able to answer more than 76.2% of the questions in a fair, relatable way and more than 66% in a good way. RER metric returns a score of 0.23 which indicates that the answers are reasonably coherent.

**Table 6:** Examples of questions and CA system responses

Question	Answer
Is it safe to take melatonin?	Here is what I found about pure crystalline Melatonin: Melatonin may cause drowsiness. Patients should not drive or operate heavy machinery until familiar with the effects of melatonin
Are there any dangerous side effects that anyone has experienced with the supplement milk thistle?	The Blessed Thistle preparation has adverse effects like Eye disorders
Are there any proven bene- fits to taking shark cartilage?	Shark Cartilage is effective for Degenerative Polyarthritis

## Discussion

This paper describes the first CA system for answering DS questions to consumers. Developing specialized domain restricted CA systems is a challenging task and especially for a largely unexplored domain such as DS. In this paper, we presented our work on building a robust CA for answering real-world questions on DS collected from Yahoo! Answers.

Question understanding can be formulated into 2 subtasks: question type classification and named entity recognition. For question type classification task, despite restricted dataset, deep learning methods outperformed conventional machine learning models. Specifically, among different deep learning methods, CNN based question type classification gave best results. This is primarily because a CNN architecture is able to incorporate some local context. CNN outperforms shallow models such as SVM, and Naive Bayes because CNN can provide semantically meaningful feature representations as compared to shallow learning models [40]. But, CNN is also more accurate than an LSTM, especially during the feature extraction step because a 1D CNN [29] processes text as a one-dimensional image and is used to capture the latent associations between neighboring words (spatial context), in contrast with LSTMs, which process each word in a sequential pattern [41, 42]. Thus, many works like [41] exploit an ensemble model of CNN and LSTM.

For our second task-NER, it is unsurprising that a single CRF layer achieves the best results given our limited data points. Comparative performance of CRF and LSTM does not follow a particular trend but is dependent on several crucial factors, mainly size and type of training data which is similar to factors we discussed just now. In [43], LSTM with CRF performs reasonably well by itself, although, the CRF model seems to perform better. As shown in studies [44, 45], LSTM approach performs best. Also, there is no conclusive evidence that a combined use of LSTM and CRF systems in hybrid or ensemble models will always outperform vanilla CRF or LSTM, such as [45]. Thus, the suitability of a model architecture in most cases is use-case dependent and in our case, due to additional factors such as time constraints, the CRF approach works the best.

Both extracted question types and named entities help the CA to generate appropriate responses to a user query. Question classification output tells us which type of question we are dealing with, whereas NER module extracts relevant named entities present in the query which helps the CA to give the most suitable result from the knowledge base. Our end-to-end evaluation method aims to ensure the feasibility of the whole system. Query response was evaluated using average score and succ@i and robustness of our conversational system as a whole was evaluated using RER. The system achieves an overall accuracy of 81% and an average score of 1.82 with succ@3+ score of 76.2% and succ@2+ of 66% approximately. The succ@3+ score is greater than succ@2+ score, which is expected because as we increment the score range (grade scale1–4) probability of correct answers decreases.

Error analysis with respect to the RER score showed a possible explanation for a relatively higher score is discrepancy in individual reviewers’ preferences. Some preferred long detailed answers whereas some gave more preference to crisp responses. This was reflected in the score ranges of many answers. Also, among our test samples for a few questions, we saw wrong answers being generated by our system and were rated 1 by the reviews. For example: for the question “is the caffeine and guarana in it making me pee?”, a wrong response was generated: “Guarana is effective for Psychiatric problem”. These wrong answers were mainly a result of error propagation from either the NER step or the question classification step. For example, our system was unable to identify cinnamon sticks as an entity in the question “does eating cinnamon sticks really get rid of a uti?” and consequently wrongly answered the question. Similarly, for the first example of caffeine and guarana, our system was unable to identify either of the entities and also had inaccurate question classification. Thus, both these factors contributed towards a higher RER on average but in totality, the system still remained reasonably coherent in its performance.

Our work has several limitations. The sample size is still very limited. The domain coverage is not quite wide and focuses only on three major relations from iDISK. We still need to find a more intensive way to categorize the questions' types into not the 8 predefined classes but a mix of them as well so that layered questions which span more than single question types can be answered more reliably.

We will also expand hierarchical components of intents and entities further along with respective annotated samples so that we can increase generalization of questions within the confines of our DS domain. More comprehensive experimentation (with more data) is required to evaluate the generated responses and their refinement into more human-like answers. We should also incorporate user feedback when the system is fully developed. The future scope of this work is to explore the development of word embeddings specific to DS domain and introduce voice-based input for the conversational system. Unlike many other systems using retrieval-based methods, we leverage backend knowledge base, as it provides high-quality and easy-to-query knowledge. In addition, DS information is disparate through the Internet, and the availability and quality of question-answer pairs are uncertain.

## Conclusion

In this study, we have developed a CA system for answering user questions about DS use using a DS knowledge base and MindMeld framework. We developed two components for understanding the natural language questions: question type classifier (F1: 0.810) and named entity recognition (F1 0.850). We demonstrated that it is feasible to integrate developed models into the MindMeld framework and answer most questions accurately.

## Supplementary Information

**Additional file 1**. Methods for questions understanding module.

## Data Availability

The dataset generated and analysed during the current study are available in the GitHub repository, https://github.com/zhang-informatics/Supplement_Conversational_agent

## Abbreviations

AI: Artificial intelligence
API: Application programming interface
Bi-LSTM: Bi-directional LSTM
BIO: Beginning, inside, out
CA: Conversation system
CRF: Conditional random fields
CNN: Convolutional neural network
DL: Deep learning
DS: Dietary supplement
KB: Knowledge-base
ML: Machine learning
NER: Named entity recognition
NLP: Natural language processing
NM: Natural medicine
RER: Response error rate
QU: Question understanding
XMI: XML metadata interchange
