# Supplementary material for: A conversational agent system for dietary supplements use
Source: BMC Med Inform Decis Mak. 2022 Jul 7;22(Suppl 1):153. doi: 10.1186/s12911-022-01888-5 (PMC9264487; doi:10.1186/s12911-022-01888-5)
Supplement: Supplementary file 1 — Additional file 1. Methods for questions understanding module. [file 12911_2022_1888_MOESM1_ESM.docx]

**SUPPLEMENTARY MATERIAL**

Here we include the Question Understanding module's two main tasks: *Question Classification* and *Named Entity Recognition’s* experimental supplementary details.

2.3.1 Question Classification

We experimented with a set of parameters, including the number of filters (ie, 64,128, 256, and 512), and filter sizes (ie, 1,2,3,5, and 7). The optimal hyperparameters are as follows: positional embedding dimension of 300, filter sizes of 1-7 and 128 filters for each size. The dropout rate is 0.1, 0.2. Weighted precision, recall, and F1score were used as the evaluation metrics. Due to the limited sample size, we used 10-fold cross validation to evaluate the question classification component.

2.3.2 Named Entity Recognition

The BiLSTM model comprised 4 layers including a BiLSTM layer and a last CRF layer. Different numbers of hidden units in the Bi-LSTM layer were tested (ie, 30, 64, 128) and the optimal hidden size was set as 30. The ReLU activation, dropout of 0.1 and Adam optimiser were used for training the model. Optimal hyper-parameters for best performing vanilla CRF model - c1, c2 as 0.1, 32 batch size,maximum iteration - 100. Features used to train the CRF model include word suffix, part-of-speech (POS) tags, the POS tags of the nearby words (1 word before and 1 word after), etc. Due to the limited sample size, we used 10-fold cross validation to evaluate the question classification component. Weighted precision, recall, F1 and accuracy metrics were used for evaluation.
